# Autophagy and the primary cilium in cell metabolism: What’s upstream?

**DOI:** 10.3389/fcell.2022.1046248

**Published:** 2022-11-09

**Authors:** Aurore Claude-Taupin, Nicolas Dupont, Patrice Codogno

**Affiliations:** Institut Necker Enfants Malades (INEM), INSERM UMR-S1151, CNRS UMR-S8253, Université Paris Cité, Paris, France

**Keywords:** cilium, cell signaling, macroautophagy, mechanical forces, mitochondria

## Abstract

The maintenance of cellular homeostasis in response to extracellular stimuli, i.e., nutrient and hormone signaling, hypoxia, or mechanical forces by autophagy, is vital for the health of various tissues. The primary cilium (PC) is a microtubule-based sensory organelle that regulates the integration of several extracellular stimuli. Over the past decade, an interconnection between autophagy and PC has begun to be revealed. Indeed, the PC regulates autophagy and in turn, a selective form of autophagy called ciliophagy contributes to the regulation of ciliogenesis. Moreover, the PC regulates both mitochondrial biogenesis and lipophagy to produce free fatty acids. These two pathways converge to activate oxidative phosphorylation and produce ATP, which is mandatory for cell metabolism and membrane transport. The autophagy-dependent production of energy is fully efficient when the PC senses shear stress induced by fluid flow. In this review, we discuss the cross-talk between autophagy, the PC and physical forces in the regulation of cell biology and physiology.

## Introduction

The recent years have witnessed the importance of the role of autophagy in cell metabolism (Kaur and Debnath, 2015; Lahiri et al., 2019; Levine and Kroemer, 2019; Poillet-Perez and White, 2019; He, 2022). Beyond the recycling of nutrients from the lysosomal compartment to fuel different metabolic pathways (Ballabio and Bonifacino, 2020), recent research has highlighted the function of selective forms of autophagy, such as lipophagy (degradation of lipid droplets) (Schulze et al., 2017) and mitophagy (partial or total degradation of mitochondria) in metabolism (Onishi et al., 2021). The impact of autophagy on cell metabolism assumes different forms in mammals: Macroautophagy (see next paragraph), microautophagy (uptake of cytoplasmic material at the surface of lysosomes or endosomes) [reviewed in (Oku and Sakai, 2018; Tekirdag and Cuervo, 2018; Schuck, 2020)] and chaperone-mediated autophagy (CMA), which allows the selective transport of proteins containing a KFERQ motif into the lysosomal lumen in a manner dependent on chaperones and on the lysosomal membrane protein LAMP2A [Reviewed in (Kaushik and Cuervo, 2018)]. As mentioned above, macroautophagy (hereafter referred to as autophagy) contributes to metabolism mostly *via* different aspects: by providing nutrients (amino acids, nucleosides, fatty acids and monosaccharides) for different metabolic pathways (Mortimore and Poso, 1987; Yim and Mizushima, 2020), by generating fatty acids (lipophagy) available for lipid synthesis or for fueling oxidative phosphorylation (Miceli et al., 2020; Xie et al., 2020; Zhang et al., 2022) and finally, by acting in the quality control of mitochondria (mitophagy). Thus, autophagy contributes in adapting cell metabolism to the cell program, e.g., by reorienting a cell from oxidative phosphorylation to glycolysis or by controlling the levels of reactive oxygen species generated by mitochondria (Pickles et al., 2018). In turn, autophagy is controlled by nutrients and metabolites, such as acetyl-CoA and α-ketoglutarate (Pietrocola et al., 2015; Baracco et al., 2019). CMA, which is stimulated by starvation, contributes to glucose metabolism by regulating the levels of many enzymes engaged in glycolysis and the TCA cycle (Schneider et al., 2014). CMA also contributes to lipid metabolism by degrading perilipins at the surface of lipid droplets (Kaushik and Cuervo, 2015), a prerequisite for lipophagy. In addition, several enzymes involved in lipogenesis are substrates for CMA (Tasset and Cuervo, 2016). Although microautophagy was first recognized in hepatocytes under starvation (Mortimore and Poso, 1984; Mortimore et al., 1988), it was characterized in yeast, where bulk and various forms of selective microautophagy were identified [reviewed in (Oku and Sakai, 2018; Schuck, 2020)]. In mammals, endosomal-microautophagy (Tekirdag and Cuervo, 2018) and Mitochondrial-derived Vesicle (MDV)-micromitophagy are the best studied forms of microautophagy (Lemasters, 2014). MDV-microautophagy, along with mitophagy, contribute to the quality control of mitochondria (Pickles et al., 2018).

Many signaling pathways emanate from the primary cilium and among these, several are engaged in the regulation of metabolism (Pala et al., 2017; Anvarian et al., 2019; Avalos et al., 2022; Yang et al., 2022a). For example, an interplay between the Hedgehog and mTORC1 pathways exist (Larsen and Moller, 2020), and both are regulated by the primary cilium. Also, cAMP produced by the type III adenylyl cyclase in neuronal primary cilium contributes to energy balance (Yang et al., 2022b), and mutations in the ciliary melanocortin 4 receptor in paraventricular hypothalamic neurons give rise to obesity (Lee et al., 2022). Moreover, a defective primary cilium impairs adipose-derived mesenchymal stem cell (ASCs) functions (Strong et al., 2015; Louwen et al., 2018) that are responsible for cell renewal, spontaneous repair and immunomodulation in adipose tissue, suggesting that obesity could be considered as a secondary form of ciliopathy (Ritter et al., 2018).

The specific aim of the present review is to present the importance of the cross-talk between autophagy and the primary cilium in the control of metabolism and also to highlight stimuli and signaling pathways that emanate from the primary cilium to regulate autophagy. Readers interested in a deeper discussion on the cross-talk between the primary cilium and autophagy and/or on the role of primary cilium in regulating proteostasis can consult recent reviews on these topics (Orhon et al., 2015; Pampliega and Cuervo, 2016; Boukhalfa et al., 2019; Wiegering et al., 2019; Morel et al., 2021; Morleo et al., 2022; Senatore et al., 2022).

## The autophagic pathway

Autophagy is characterized by the formation of a double-membrane-bound autophagosome (Mizushima et al., 2008; Boya et al., 2013). The autophagosome, which originates from a membranous structure named the phagophore, sequesters fractions of the cytoplasm, in a selective or non-selective manner, to deliver them to the lysosome (Khaminets et al., 2016) [Readers interested in the origin of the phagophore can consult recent reviews (Deretic and Lazarou, 2022; Hu and Reggiori, 2022)]. Autophagy is regulated by Autophagy-related (ATG) proteins and associated factors (Nakatogawa et al., 2009; Mizushima et al., 2011). Initiation of autophagosome formation is triggered by changes in cell nutritional levels or by different stress situations (Levine and Kroemer, 2019). Autophagy stimulation is typically dependent on the downregulation of mTORC1 kinase activity leading to stimulation of the ULK complex, which consists of the serine/threonine kinase ULK1 or ULK2 (mammalian homologs of yeast Atg1), ATG101, ATG13 and a 200 kDa focal adhesion kinase family-interacting protein (FIP200, also called RB1CC1) (Kawabata and Yoshimori, 2020; Nishimura and Tooze, 2020). The growth of the autophagosome membrane depends on ATG9A-containing vesicles. Indeed, ATG9A, which harbor a scramblase activity, binds to ATG2, a factor able to transfer phospholipids from the ER to the phagophore in formation (Sawa-Makarska et al., 2020). This interaction is important for expanding the phagophore (Maeda et al., 2020; Chang et al., 2021). The ULK complex stimulates the class III phosphoinositide 3-kinase (PI3KIII) complex. The core PI3KIII complex is composed of the kinase VPS34 (encoded by *PIK3C3* in mammals), VPS15, ATG14L1 and Beclin1 (homolog of yeast Atg6) (Kawabata and Yoshimori, 2020; Nishimura and Tooze, 2020). VPS34 allows the formation of phosphatidylinositol 3-phosphate (PI3P) to recruit the PI3P-interacting proteins WIPI2B and ATG16L1, followed by the interaction of the ATG12–ATG5 ubiquitin-like conjugate to form a complex with ATG16L1. The ATG12–ATG5-ATG16L1 complex triggers the conjugation of ATG8 family LC3/GABARAP proteins to phosphatidylethanolamine, which will be integrated in the autophagic membrane. During the different forms of selective autophagy, the ATG8 family proteins can bind to cargo receptors, including p62 (SQSTM1), NDP52 and optineurin, which harbor LC3-interacting region (LIR) motifs (Johansen and Lamark, 2020; Gubas and Dikic, 2022). After closure and maturation, the autophagosome can deliver its content to endosomes or lysosomes, in a process regulated by several proteins, including small Rab GTPases and SNAREs, to induce the degradation of the autophagy cargo (Zhao et al., 2021). The autophagic pathway is also controlled at transcriptional and epigenetic levels (Fullgrabe et al., 2014). Of note, the inhibition of mTORC1 also allows for the nuclear translocation of the transcription factor TFEB, which controls lysosomal biogenesis as well as the expression of several key autophagy genes (*SQSTM1*, *WIPI1*, *WIPI2*, *MAP1LC3B*, *ATG9B*) (Napolitano and Ballabio, 2016).

## The primary cilium

Cilia are microtubule-based structures present at the surface of different cell types. Conventionally, cilia which are motile or non-motile, are classified by their microtubule structure and motility (Satir and Christensen, 2007). Motile cilia (MC) is a beating organelle which generate fluid flow. MC are present on the surface of epithelial cells of the ependyma and in the brain as well as on epithelial cells lining the airways and reproductive tracts. In contrast to MC, primary cilia are solitary organelles found in almost all cell types, with some exceptions such as some cells of the lymphoid and myeloid lineage and other cells like hepatocytes (Finetti et al., 2014; Douanne et al., 2021). From a structural point of view, they consist of an axoneme of nine doublet microtubules growing from a basal body that is coming from one of the centrioles of the centrosome (the mother centriole) (Malicki and Johnson, 2017). Of note, ciliogenesis and cell division are mutually exclusive. A ciliary subdomain highly organized called transition zone is crucial for gating the entry of cytosolic factors into the axoneme (Satir and Christensen, 2007; Garcia-Gonzalo and Reiter, 2012). Primary cilia is a sensory non-motile organelle playing a role during embryonic development, olfaction, vision, and mechanotransduction (Satir and Christensen, 2007; Goetz and Anderson, 2010). They respond to different stimuli such as mechanical stimuli [for example, shear stress that may lead to cilia bending (Ferreira et al., 2019)]and chemical stimuli [e.g., specific ligand, growth factor, hormone or morphogen recognition (Satir and Christensen, 2007; Goetz and Anderson, 2010)]. Various signaling pathways emanate from this antenna, such as the Hedgehog and Wnt pathways, the platelet-derived growth factor (PDGF) pathway (Anvarian et al., 2019; Nachury and Mick, 2019). Intraflagellar transport (IFT) complexes are crucial for the ciliogenesis (Nakayama and Katoh, 2018; Nachury and Mick, 2019; Pigino, 2021). While the IFT-B complex consists of 16 proteins, which together with the motor protein kinesin 2, are involved in the transport of proteins to the tip of the primary cilium, the IFT-A complex, which is composed of six factors, contributes to the protein transport from the tip to the basal body of the primary cilium in a dynein-dependent manner (Nakayama and Katoh, 2018). Abnormalities of cilia structure or/and function lead to human diseases known as ciliopathies (Braun and Hildebrandt, 2017; Anvarian et al., 2019).

## Primary cilium and autophagy in cell metabolism regulation

**FIGURE 1:**
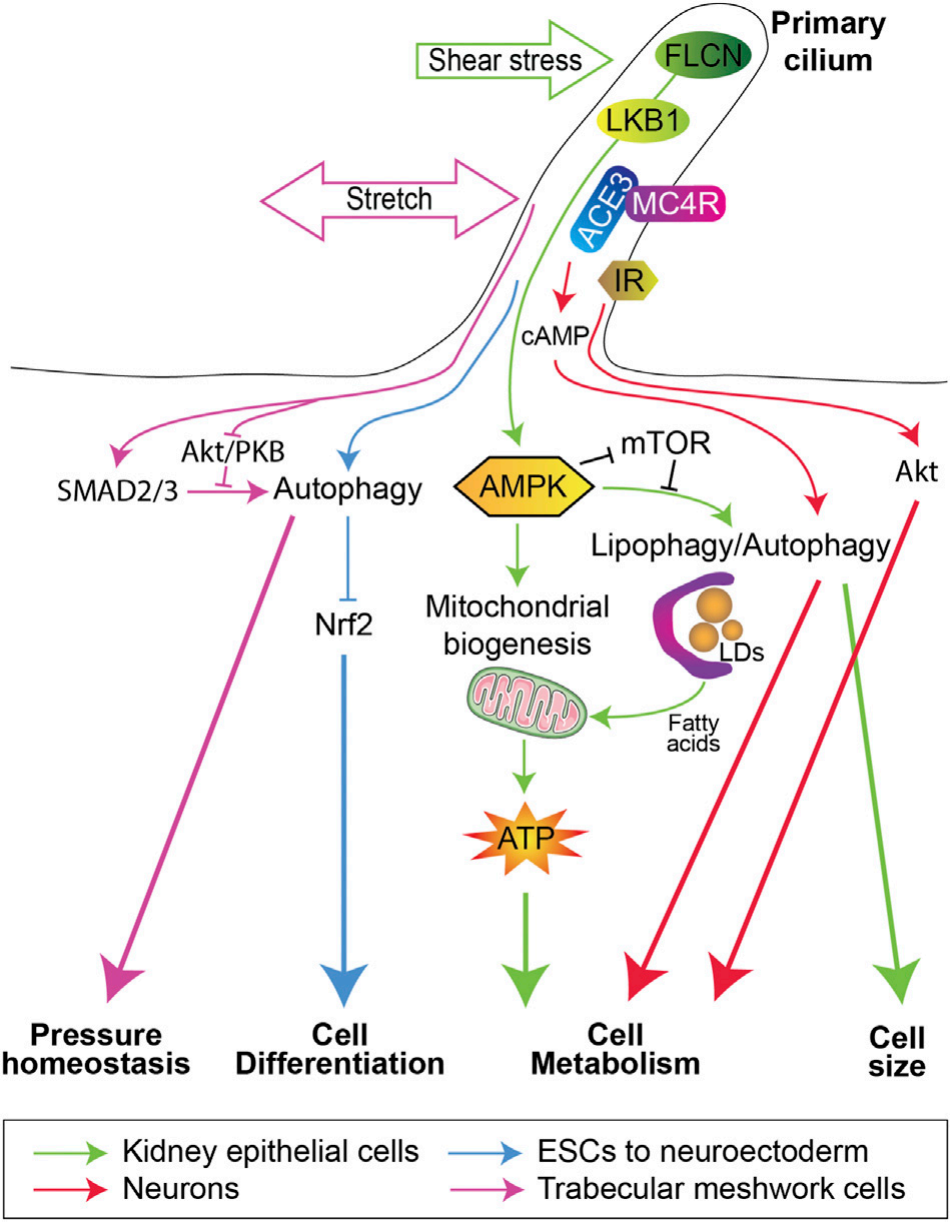
Primary cilium and autophagy in the regulation of cell fate. Shear stress in kidney epithelial cells (Green arrows) activates the folliculin (FLCN)/LKB1/AMPK/mTORC1 cascade to induce on one hand mitochondria biogenesis and on the other hand autophagy/lipophagy to regulate cell size and to produce fatty acids from lipid droplets (LDs) degradation, which will be used by mitochondria for ATP synthesis. This energy production favorizes the kidney epithelial cells metabolic reprogramming to control many activities, e.g., glucose reabsorption and gluconeogenesis. Stretch in trabecular meshwork cells (Purple arrows) activates the SMAD2/3 pathway and inhibits the Akt/PKB signaling, to positively regulate autophagy and control intraocular pressure homeostasis. Neuronal primary cilium (Red arrows) can regulate cell metabolism by the melanocortin 4 receptor (MC4R) or Insulin receptor (IR), which control cAMP production *via* type III adenylyl cyclase (ACE3) and Akt signaling, respectively. In embryonic stem cells (Blue arrows), a primary cilium-dependent inhibition of nuclear factor erythroid-related factor 2 (Nrf2) promotes their differentiation towards a neuroectodermal fate.

The first evidence of a primary cilium-dependent activation of autophagy was originally described in cells deprived of serum and/or nutrients (Pampliega et al., 2013; Tang et al., 2013). Subsequently, fluid flow (shear stress) has been shown to induce autophagy in a primary cilium-dependent manner in kidney proximal tubule epithelial cells (PTEC), thus highlighting a physiological role for primary cilium-dependent autophagy. Indeed, in PTEC, primary cilia are located at the apical membrane facing the urinary flow and regulates the cell size upon shear stress (Boehlke et al., 2010). This process is dependent on the stimulation of a pool of AMP-activated protein kinase (AMPK) at the base of the cilium (basal body), downstream of liver kinase B1 (LKB1) (Boehlke et al., 2010). Afterwards, we demonstrated the LKB1/AMPK-dependent activation of autophagy plays a crucial role in the regulation of cell size in kidney epithelial cells subjected to shear stress (Orhon et al., 2016). In fact, two signaling pathways dependent on AMPK come from the primary cilium (Miceli et al., 2020). On one hand, the fluid flow-dependent activation of AMPK upregulates the mitochondrial mass by controlling the expression of two master regulators of mitochondrial protein expression: peroxisome proliferator-activated receptor-gamma coactivator alpha (PGC1α) and mitochondrial transcription factor A (TFAM). On the other hand, AMPK induces a form of selective autophagy that degrades lipid droplets, called lipophagy (Singh et al., 2009a; Roberts and Olzmann, 2020), to generate free fatty acids used as substrates in mitochondria (Walch et al., 2015; Ogasawara et al., 2020; Rodriguez et al., 2021). Together, mitochondrial biogenesis and lipophagy in PTEC allow ATP production to supports energy consuming cellular processes, such as glucose reabsorption and gluconeogenesis (Miceli et al., 2020) (Figure 1). It is worth noting that under physiological conditions, PTEC contain numerous mitochondria to produce high levels of ATP in a manner dependent on fatty acid oxidation (FAO) (Bhargava and Schnellmann, 2017), supported by the lack of lipid droplets (Minami et al., 2017; Yamamoto et al., 2017). However, during fasting periods (Minami et al., 2017) or pathological situations such as acute kidney injury (AKI) (Li et al., 2009; Zhang et al., 2018), lipid droplets can be observed. Accordingly, it has been reported that tubular injury upon AKI is associated with the downregulation of PGC1α expression, mitochondrial and FAO dysfunction (Zhan et al., 2013; Zuk and Bonventre, 2016; Zhang et al., 2018). Moreover, in cisplatin-induced injury, the length of primary cilium is shortened and associated with reduced autophagy levels and mitochondrial dysfunction (Fujii et al., 2021; Wang et al., 2021). Defective FAO (Menezes et al., 2016; Warner et al., 2016), altered mitochondrial oxidative phosphorylation (Hajarnis et al., 2017; Ishimoto et al., 2017) and impairment of autophagy (Belibi et al., 2011; Rowe et al., 2013) have also been observed in cells lining renal cysts in a mouse model of autosomal dominant polycystic kidney disease (ADPKD) and in renal tissue from ADPKD patients (Padovano et al., 2018). ADPKD is mainly associated with mutations in the *PKD1* or *PKD2* genes, encoding the ciliary proteins polycystin-1 and -2 (PC1 and PC2), respectively (Hughes et al., 1995; Mochizuki et al., 1996). It can thus be speculated that a loss of primary cilium-dependent stimulation of autophagy could contribute to the development and/or early stages of these ciliopathies. Moreover, the maintenance of tissue homeostasis dependent on autophagy and mitochondrial biogenesis is not only restricted to the renal tubules. Indeed, in endothelial cells, fluid flow can also support mitochondrial biogenesis and ATP production (Kim et al., 2015; Wu et al., 2018; Yamamoto et al., 2018). However, the primary cilium does not function upstream of autophagy in endothelial cells submitted to high shear stress (Vion et al., 2017) and its role upstream of mitochondrial biogenesis has not been documented.

Other mechanosensors can transduce shear stress, as represented by microvilli-dependent formation of vacuoles in epithelial intestinal cells (Kim et al., 2017), which lack primary cilia. The formation of these vacuoles depends on a subset of ATG (ATG5 and LC3, independent of Beclin 1). This work showed that microvilli transduce shear stress independently of the primary cilium to initiate an ATG-dependent vacuolar process. The authors speculate that these vacuoles could be responsible for the degradation or secretion of macronutrients by intestinal enterocytes.

Stem cell renewal and differentiation are dependent on the functioning of the primary cilium to initiate many signaling pathways (Yanardag and Pugacheva, 2021; Shimada and Kato, 2022; Bodle and Loboa, 2016) and on autophagy, which contributes to proteostasis and metabolic control *via* mitophagy (Boya et al., 2018). Recently, the coordinated contributions of autophagy and the primary cilium to the differentiation of embryonic stem cells (ESCs) has been shown (Jang et al., 2016). The primary cilium has indeed been demonstrated to emerges from ESCs after induced lineage specification and triggers autophagy. This leads to the inactivation of nuclear factor erythroid-related factor 2 (Nrf2), upregulating the transcriptional stimulation of the pluripotency proteins OCT4 and NANOG and directing ESCs towards a neuroectodermal fate (Jang et al., 2016) (Figure 1). In summary, autophagy is important to fulfil the energy requirements for ESCs remodeling and metabolic changes.

While not directly related to the topic of the review, it is important to keep in mind that a complex crosstalk exist between the primary cilium and the ubiquitin proteasome system (UPS) (Orhon et al., 2015; Pampliega and Cuervo, 2016; Boukhalfa et al., 2019; Wiegering et al., 2019; Morel et al., 2021; Morleo et al., 2022; Senatore et al., 2022) and between autophagy and the UPS (Kwon and Ciechanover, 2017; Pohl and Dikic, 2019). The UPS plays an important role for controlling ciliogenesis and disassembly of the primary cilium (Kim et al., 2010; Wheway et al., 2015). As an example of that, the UPS regulates ciliogenesis by controlling the expression of the centriole and centriolar satellite protein OFD1 (Senatore et al., 2021). Interestingly, the activation of UPS by the SARS-CoV2 protein ORF10 increases the degradation of ciliary proteins, thus impairing ciliogenesis (Wang et al., 2022). In addition, the UPS can turn off signaling pathways initiated by the primary cilium (Gerhardt et al., 2016; Wiegering et al., 2019; Szymanska et al., 2022). Whether the primary cilium-UPS axis operates downstream of mechanosensors to regulate metabolism remains to be investigated.

## What is the upstream control of the cross-talk between autophagy and primary cilium

Several signaling pathways could stimulate autophagy in a primary cilium-dependent manner and vary according to cell type and stimuli. For example, it was reported that induction of canonical ciliary Hedgehog signaling (activation of Smo and transcription factor Gli2) was crucial to trigger primary cilium-dependent autophagy upon serum starvation in kidney epithelial cells, fibroblasts and neurons (Pampliega et al., 2013). Recently, it has been reported that a non-canonical hedgehog signaling pathway (without stimulation of Gli transcription factors) initiates ciliogenesis and autophagy (Akhshi and Trimble, 2021). Indeed, on one hand, Smo activates the LKB1-AMPK axis to activate autophagy and early stages of ciliogenesis and on the other hand a heterotrimeric Gi pathway to promote ciliogenesis. Interestingly, the cross-talk between the primary cilium and autophagy *via* the Gli2 transcription factor modulates cell cycle re-entry (Hsiao et al., 2018).

Upon shear stress, autophagy is induced in kidney epithelial in a manner dependent on the mTORC1/AMPK pathway, as mentioned in the preceding paragraph, but also by PC2, a primary cilium-associated calcium channel (Orhon et al., 2016). In contrast to the mTORC1/AMPK dependent autophagy, PC2-dependent autophagy is not important for cell size regulation. However, based on recent findings, PC2-dependent autophagy could be part of a mechanism acting during kidney repair after structural changes (Dong et al., 2021). Fluid flow senses by the primary cilium triggers LKB1 activation, located in the axoneme, thus allowing the stimulation of the mTORC1/AMPK pathway (Boehlke et al., 2010; Orhon et al., 2016). In this system, folliculin promotes the recruitment of LKB1 at the primary cilium, participates in the regulation of AMPK activity, autophagy and cell size (Zemirli et al., 2019). Moreover, patients suffering from a ciliopathy called Birth-Hogg-Dubbé syndrome present heterozygous mutations in the folliculin gene, suggesting that the activation of autophagy could be an important factor in maintaining cellular homeostasis in a primary cilium-dependent manner (Luijten et al., 2013). Interestingly, LKB1 has been shown to partner with the PC1 mechanosensor in the primary cilium (Viau et al., 2018). PC1, in contrast to PC2, controls kidney epithelial cell size (Viau et al., 2020). Thus, it is tempting to hypothesize that PC1 is the membrane sensor upstream of the LKB1/AMPK axis to control autophagy and cell size.

Similarly, primary cilium-dependent inhibition of mTORC1 stimulates autophagy in chondrocytes in response to cyclic tensile strain (Xiang et al., 2019). Mechanical stretch sensing by the primary cilium can also regulate autophagy in the trabecular meshwork to regulate the intraocular pressure (Shim et al., 2021), in a process dependent on the SMAD2/3 pathway and inhibited by Akt/PKB (Figure 1). The protein RPGRIP1L, encoded by the *Rpgrip1l/Ftm/Mks5/Nphp8* (Rpgrip1-like) gene and mutated in patients suffering from deadly ciliopathies (Arts et al., 2007; Delous et al., 2007), is located in the primary cilium transition zone and controls autophagy by inhibiting mTOR signaling (Struchtrup et al., 2018). Interestingly, RPGRIP1L regulates autophagy and the proteasome pathway *via* two independent signaling pathways (Gerhardt et al., 2015; Struchtrup et al., 2018).

Regarding the molecular events required for autophagosome formation following primary cilium sensing, it has been reported that many ATGs (except ULK1 and Beclin1) can be recruited at the primary cilium upon serum starvation or shear stress (Pampliega et al., 2013; Boukhalfa et al., 2020). In contrast to bulk autophagy initiation, where the ULK and PI3KIII complexes are essential for PI3P formation, autophagy induction in kidney epithelial cells submitted to fluid flow has been shown to be dependent on PI3P synthesis by the class II PI3Kα (PI3K-C2α) (Boukhalfa et al., 2020). Importantly, the PI3K-C2α kinase controls ciliogenesis and is relocated at the primary cilium upon shear stress (Franco et al., 2014; Boukhalfa et al., 2020). Moreover, PI3KC2α ± mice exhibit defects in autophagic stimulation and cell-size regulation in kidney tubular cells (Boukhalfa et al., 2020). Complementary studies are however needed to decipher the molecular signaling necessary to recruit and regulate the activity of PI3K-C2α at the primary-cilium upon shear stress. This would help to understand if the phagophore is directly produced at the vicinity of the primary cilium. How AMPK controls autophagy in the absence of ULK1/2 and Beclin1 is still an open question. An epigenetic regulation of autophagy by AMPK cannot be excluded in this setting (Li et al., 2017).

## Conclusion

The past several years have illuminated the cross-talk between the primary cilium and the two major cellular catabolic pathways, i.e., the autophagic pathway and the ubiquitin proteasomesystem (UPS) (Orhon et al., 2015; Pampliega and Cuervo, 2016; Boukhalfa et al., 2019; Wiegering et al., 2019; Morel et al., 2021; Morleo et al., 2022; Senatore et al., 2022). Many studies have demonstrated that autophagy is engaged in the regulation of ciliogenesis by controlling the expression of factors such as IFT20 (Pampliega et al., 2013), OFD1 (Tang et al., 2013), MYH9/myosin 2 (Yamamoto et al., 2021), CP110 (Liu et al., 2021) or Kif19A (Arora et al., 2020). As such, the word “ciliophagy” has been adequately introduced to designate the selective degradation of ciliary proteins by the autophagic pathway (Cloonan et al., 2014). In addition, the ciliary proteins IFT20, OFD1 (Finetti et al., 2021; Morleo et al., 2021) and ATG16L1 (Boukhalfa et al., 2021) are engaged in the regulation of autophagy and ciliogenesis, respectively.

As discussed in this review, a major outcome of the regulation of the cross-talk between the primary cilium and autophagy is the contribution to metabolism, including the fitness of mitochondria. However, it can be surmised that this cross-talk has consequences beyond the regulation of cell metabolism, such as cell differentiation and major physiological processes such as memory acquisition. In line with this assumption, both the primary cilium and autophagy are engaged in white adipocyte differentiation (Singh et al., 2009b; Zhang et al., 2009; Hilgendorf et al., 2019; Zhang et al., 2021) and are known to be involved in hippocampal memory acquisition (Glatigny et al., 2019; Jovasevic et al., 2021; Bashford and Subramanian, 2022). Whether or not the primary cilium and the autophagic pathway communicate during these processes is still an open question. Undoubtedly, future studies will illuminate the importance of the cross-talk between autophagy and the primary cilium in biological and physiological responses to the environment.
